# Assessment of Quality of Life Using the Kidslife Scale in Individuals With Cornelia de Lange Syndrome

**DOI:** 10.7759/cureus.57378

**Published:** 2024-04-01

**Authors:** Laura Trujillano, Ariadna Ayerza-Casas, Beatriz Puisac, Ana Latorre-Pellicer, María Arnedo, Cristina Lucia-Campos, Marta Gil-Salvador, Ilaria Parenti, Frank J Kaiser, Feliciano J Ramos, Javier Trujillano, Juan Pié

**Affiliations:** 1 Department of Clinical and Molecular Genetics, Vall d'Hebron Hospital, Barcelona, ESP; 2 Medicine Genetics Group, Vall Hebron Research Institute, Barcelona, ESP; 3 Unit of Paediatric Cardiology, Service of Paediatrics, Hospital Universitario Miguel Servet, Zaragoza, ESP; 4 Unit of Clinical Genetics and Functional Genomics, Department of Pharmacology-Physiology, School of Medicine, Universidad de Zaragoza, CIBERER-GCV02 and IIS-Aragon, Zaragoza, ESP; 5 Institute for Human Genetics, University Hospital Essen, University of Duisburg-Essen, Essen, DEU; 6 Essen Center for Rare Diseases, University Hospital Essen, Essen, DEU; 7 Unit of Clinical Genetics, Department of Paediatrics, Service of Paediatrics, Hospital Clínico Universitario Lozano Blesa, Zaragoza, ESP; 8 Department of Intensive Care Medicine, Hospital Universitario Arnau de Vilanova de Lleida, Lleida, Spain; Institut de Recerca Biomèdica de Lleida, Lleida, ESP

**Keywords:** clinical features, kidslife, intellectual disability, quality of life, cornelia de lange syndrome

## Abstract

Background: Cornelia de Lange syndrome (CdLS) is a rare polymalformative genetic disorder with multisystemic involvement. Despite numerous clinical and molecular studies, the specific evaluation of the quality of life (QoL) and its relationship with syndrome-specific risk factors has not been explored.

Methods: The QoL of 33 individuals diagnosed with CdLS, aged between 4 and 21 years, was assessed using the Kidslife questionnaire. Specifically, the influence of 14 risk factors on overall QoL and 8 of its domains was analyzed.

Results: The study revealed below-median QoL (45.3 percentile), with the most affected domains being physical well-being, personal development, and self-determination. When classifying patients based on their QoL and affected domains, variants in the NIPBL gene, clinical scores ≥11, and severe behavioral and communication issues were found to be the main risk factors.

Conclusions: We emphasize the need for a comprehensive approach to CdLS that encompasses clinical, molecular, psychosocial, and emotional aspects. The "Kidslife questionnaire" proved to be a useful tool for evaluating QoL, risk factors, and the effectiveness of implemented strategies. In this study, we underscore the importance of implementing corrective measures to improve the clinical score. Furthermore, we highlight the necessity of applying specific therapies for behavioral problems after ruling out underlying causes such as pain or gastroesophageal reflux and implementing measures that facilitate communication and promote social interaction.

## Introduction

Cornelia de Lange syndrome (CdLS) (OMIM #122470, #300590, #610759, #614701, #300882) is a congenital multisystemic disorder with an estimated incidence of one in every 10,000 to 30,000 live births. It is a genetic disease caused by pathogenic variants in genes encoding structural and regulatory components of the cohesin complex, often found in mosaic form. While variants in the NIPBL gene account for up to 70% of diagnoses [1-5], other causal genes for the disease have been identified, such as SMC1A [6], SMC3 [7], RAD21 [8], BRD4 [9], HDAC8 [10], ANKRD11 [11], and MAU2 [12].

CdLS is clinically characterized by distinct facial features, growth delay, intellectual disability, upper limb abnormalities, hypertrichosis, and dysfunctions in various body systems. Facial characteristics include microcephaly, arched eyebrows, long eyelashes, thin upper lip, and low-set ears. Limb differences can range from small hands to the complete absence of upper limbs. Additionally, affected individuals may experience complications such as intestinal malrotation, congenital diaphragmatic hernia, and hearing loss, among others. There is a spectrum of presentation, from classic to milder or "non-classic" forms [13]. Intellectual disability ranges from mild to profound, with the majority being moderate, and communicative ability is particularly affected in expressive language [14]. Behavioral disorders are common, with autistic spectrum disorder, repetitive behaviors, self-injury, anxiety, hyperactivity, and sleep problems being characteristic [15-17]. Although it has been described that behavioral impairment can have a negative impact [18], systematic studies evaluating the quality of life (QoL) of individuals with CdLS are lacking.

QoL can be defined as a multidimensional state of personal well-being that encompasses cultural properties, subjective and objective aspects, and is influenced by individual and environmental factors. Multiple questionnaires allow for studying QoL in individuals with intellectual disabilities [19-21]. In this study, the Kidslife scale [22] has been chosen.

A comprehensive understanding of QoL is essential to establish improvement strategies and specific interventions by healthcare professionals, educators, and social services. This study assesses, for the first time, the QoL in individuals with CdLS, as well as its possible association with the syndrome's clinical features.

This article was previously posted to the Research Square preprint server on February 7, 2024.

## Materials and methods

Participants

We recruited a convenient sample consisting of a homogeneous group of individuals, all of whom were members of the CdLS association. The study involved 33 individuals, including 21 females and 12 males. The inclusion criteria were as follows: (a) having a diagnosis of CdLS, (b) being a member of the CdLS national association, (c) an age between 4 and 21 years, (d) having known the evaluated person for at least six months, or (e) having had the opportunity to observe the participant over long periods in different situations.

We categorized participants according to their phenotype using the clinical criteria published in the international consensus statement on CdLS [1]. We considered a classical phenotype when the score was ≥11 and at least three of the cardinal features were present; we assigned a non-classical phenotype to those with a score of 9-10 and two cardinal features. We recommended molecular studies when the score was ≥4 and at least one cardinal feature was present. The genetic diagnosis was performed using whole-exome sequencing (WES) or a targeted NGS panel that included the genes *NIPBL *[4], *SMC1A *[6], *SMC3 *[7], *RAD21 *[8], *BRD4 *[9], *HDAC8 *[10], *ANKRD11 *[11], and *MAU2 *[12].

Procedure

We initially contacted the CdLS association to explain the project. They expressed interest in participating, so we attended the annual CdLS conference in 2021, where we provided informed consent for participation in the project and the Kidslife questionnaire to all families. Parents responded to the survey, and we addressed any emerging questions in person.

Subsequently, other member families of the association showed interest in participating in the project. We provided them with the informed consent and the Kidslife questionnaire online, and we were available to address queries remotely.

In most cases, both parents completed the survey in a single session lasting 20-30 minutes. Data were collected between October and December 2021. We identified all participants with a code to safeguard their identity and ensure data confidentiality.

Instrument

We administered the KidsLife scale [22]. This scale evaluates the eight fundamental domains of QoL for individuals with intellectual disabilities, including social inclusion, self-determination, emotional well-being, physical well-being, material well-being, rights, personal development, and interpersonal relations. Comprising 96 questions, each domain is explored through 12 questions with four possible responses, ranging from 1 to 4 (never, sometimes, often, and always), leading to a direct score.

Based on the participant's age, we adjusted the direct score for each domain to a standardized score and its corresponding percentile. The total standard score (Overall QoL) was calculated by summing up these standardized scores. This value was then transformed into the QoL Index (QoLI) or compound standard score, along with its corresponding age-adjusted percentile. The QoLI adheres to a normal distribution with a mean of 100 and a standard deviation of 15. The derived percentiles were determined using data from a sample of 1060 individuals with intellectual disabilities aged 4 to 21 years.

The Spanish version of the KidsLife Scale is available for free download online [23, 24].

Risk factors

The following data were included as potential risk factors: sex, age, affected gene, clinical score, support needs, intellectual disability, communication difficulties, behavioral impairment, gastroesophageal reflux disease (GERD), limb malformation, heart disease, epilepsy, visual problems, and hearing loss [1].

Data analysis

Values were presented as mean ± standard deviation and percentages. The sample was categorized into three groups based on the QoLI percentile (Low: < p33, Medium: p33-p66, and High: > p66). The comparison of the three QoLI groups was performed using the Mann-Whitney test or chi-square test. The correlation between risk factors and the total QoLI was assessed using Spearman’s correlation coefficient. Additionally, the Kruskal-Wallis test was applied to analyze the relationship between risk factors and different domains of QoL. A p-value less than 0.05 was considered statistically significant. Statistical analysis was conducted using IBM SPSS Statistics for Windows, Version 29 (Released 2023; IBM Corp., Armonk, New York) and the Jamovi 2.3.21 program (Jamovi Project, Sydney, Australia).

## Results

Total QOLI and domain scores in individuals with CdLS

**Figure 1:**
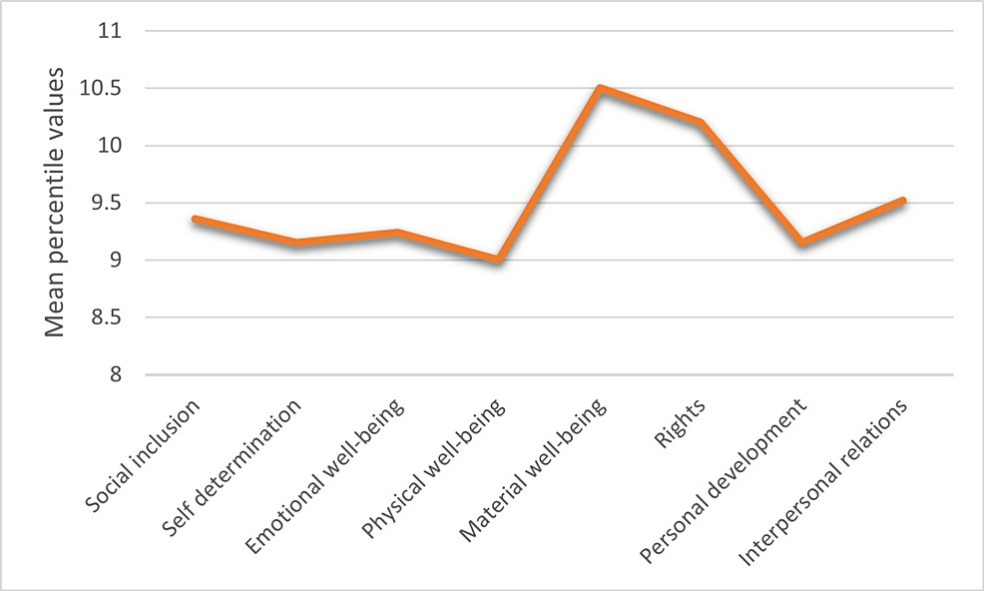
Mean percentile values obtained in each quality of life domain

The average QoLI score was 96.6 ± 17.5 (range 48 to 130), corresponding to a percentile of 45.3 ± 31.1. Among the eight domains assessed, material well-being and rights exhibited the highest standardized scores (range 1-18), with scores of 10.5 ± 3.2 and 10.2 ± 3.2, respectively, corresponding to percentiles of 59.7 ± 28.2 and 55.8 ± 30.1. Conversely, the lowest mean standardized scores were observed in the domains of physical well-being (9.0 ± 3.5, percentile 43.8 ± 30.8), personal development (9.1 ± 3.2, percentile 42.8 ± 30.4), and self-determination (9.2 ± 4.2, percentile 41 ± 35.1). Intermediate and similar scores were found in the domains of emotional well-being, interpersonal relations, and social inclusion (Table 1, Figure 1).

**Table 1:** Quality of life by domains and total in patients with CdLS (n = 33) Standardized values ​​according to the Kidslife questionnaire. x̄: mean, SD: standard deviation, CdLS: Cornelia de Lange syndrome.

Domains	Direct score (x̄ ± SD)	Standard score (x̄ ± SD)	Percentile (x̄ ± SD)	Standard score range	Standard score reference range
Social inclusion	31.2 ± 8.1	9.4 ± 3.9	45.8 ± 34.4	1–17	1–18
Self-determination	29.8 ± 8.8	9.2 ± 4.2	41 ± 35.1	2–18	1–18
Emotional well-being	39.3 ± 5.5	9.2 ± 3.1	44.7 ± 30.2	2–14	1–18
Physical well-being	40.3 ± 6.1	9.0 ± 3.5	43.8 ± 30.8	1–14	1–18
Material well-being	42.8 ± 6.1	10.5 ± 3.2	59.7 ± 28.2	1–13	1–18
Rights	41.1 ± 5.5	10.2 ± 3.2	55.8 ± 30.1	1–15	1–18
Personal development	38.5 ± 6.7	9.1 ± 3.2	42.8 ± 30.4	1–14	1–18
Interpersonal relation	37.7 ± 6.6	9.5 ± 3.4	44.9 ± 32.4	2–15	1–18
Overall quality of life (total standard score)		76.2 ± 21.2			
Quality of life index (compound standard score)		96.6 ± 17.5	45.3 ± 31.1	48–128	48–130

Study of risk factors in QoL

Significant differences were observed among the three QoLI groups (Low: QoLI < p33, Medium: QoLI p33-p66, and High: QoLI > p66) based on the affected gene (p = 0.013), clinical score (p = 0.016), and behavioral impairment (p = 0.001). Specifically, 55% (11/20) of individuals with variants in the *NIPBL* gene, 55.6% (10/18) of those with a clinical score >11 (classic phenotype), and all individuals with severe behavioral impairment (4/4) were classified in the lowest QoL group (Table 2).

**Table 2:** Sample characteristics and influence of risk factors on the quality of life index Significance level p < 0.05. SD: standard deviation; QoLI: quality of life index; ID: intellectual disability; GERD: gastroesophageal reflux disease; OPHTH: ophthalmologic.

Risk factors	Total (n = 33)	QoLI	QoLI p33-66 (n = 11)	QoLI >p66 (n = 9)	p-value
	Mean (SD)	Mean (SD)	Mean (SD)	Mean (SD)	
Age (years)	12.6 (6)	13.3 (6.8)	12.7 (4.7)	11.3 (6.5)	0.761
	n (%)	n (%)	n (%)	n (%)	
Gender					0.228
Male	12 (36.4)	5 (38.5)	2 (18.2)	5 (55.6)	
Female	21 (63.6)	8 (61.5)	9 (81.8)	4 (44.4)	
Affected gene					0.013
NIPBL	20 (60.6)	11 (84.6)	4 (36.4)	5 (55.6)	
HDAC8	4 (12.1)	1 (7.7)	0	3 (33.3)	
RAD21	3 (9.1)	1 (7.7)	2 (18.2)	0	
SMC1A	3 (9.1)	0	2 (18.2)	1 (11.1)	
Unidentified	3 (9.1)	0	3 (27.3)	0	
Clinical score					0.016
<9	9 (27.3)	0	4 (36.4)	5 (55.6)	
9-10	6 (18.2)	3 (23.1)	1 (9.1)	2 (22.2)	
>11	18 (54.5)	10 (76.9)	6 (54.5)	2 (22.2)	
Support needed					0.693
Limited	5 (15.2)	1 (7.7)	1 (9.1)	3 (33.3)	
Intermittent	7 (21.2)	2 (15.4)	3 (27.3)	2 (22.2)	
Extensive	9 (27.3)	5 (38.5)	3 (27.3)	1 (11.1)	
Generalized	12 (36.4)	5 (38.5)	4 (36.4)	3 (33.3)	
Intellectual disability					0.386
Mild	6 (18.2)	3 (23.1)	1 (9.1)	2 (22.2)	
Moderate	14 (42.4)	3 (23.1)	7 (63.6)	4 (44.4)	
Severe	13 (39.4)	7 (53.8)	3 (27.3)	3 (33.3)	
Communication difficulties					0.239
Normal	2 (6.1)	0	0	2 (22.2)	
Mild	4 (12.1)	1 (7.7)	2 (18.2)	1 (11.1)	
Moderate	15 (45-5)	5 (38.5)	5 (45.5)	5 (55.6)	
Severe	12 (36.4)	7 (53.8)	4 (36.4)	1 (11.1)	
Behavior impairment					0.001
Normal	3 (9.1)	0	0	3 (33.3)	
Mild	14 (42.4)	2 (15.4)	9 (81.8)	3 (33.3)	
Moderate	12 (36.4)	7 (53.8)	2 (18.2)	3 (33.3)	
Severe	4 (12.1)	4 (30.8)	0	0	
GERD					0.673
Normal	2 (6.1)	0	1 (9.1)	1 (11.1)	
Mild	20 (60.6)	7 (53.8)	7 (63.6)	6 (66.7)	
Moderate	7 (21.2)	4 (30.8)	1 (9.1)	2 (22.2)	
Severe	4 (12.1)	2 (15.4)	3 (18.2)	0	
Limb malformation					0.676
Normal	8 (24.2)	3 (23.1)	3 (27.3)	2 (22.2)	
Small hand/feet	20 (60.6)	7 (53.8)	6 (54.5)	7 (77.8)	
Limb reduction defect	5 (15.2)	3 (23.1)	2 (18.2)	0	
Cardiopathy					1
No	28 (84.8)	11 (84.6)	9 (81.8)	8 (88.9)	
Yes	5 (15.2)	2 (15.4)	2 (18.2)	1 (11.1)	
Seizures					1
No	27 (81.8)	11 (84.6)	9 (81.8)	6 (18.2)	
Yes	6 (18.2)	2 (33.3)	2 (33.3)	2 (33.3)	
OPHTH defects					0.555
Normal	18 (62.1)	8 (66.7)	7 (70)	3 (42.9)	
Myopia	11 (37.9)	4 (33.3)	3 (30)	4 (57.1)	
Hearing loss					0.957
Normal	21 (63.6)	7 (53.8)	8 (72.7)	6 (66.7)	
Unilateral	4 (12.1)	2 (15.4)	1 (9.1)	1 (11.1)	
Bilateral	8 (24.2)	4 (30.8)	2 (18.2)	2 (22.2)	

In the correlation analysis between the total QoLI and different risk factors, we observed an inverse correlation between QoLI and the clinical score (p < 0.01), communication problems (p < 0.05), and behavioral impairment (p < 0.05) (Table 3).

**Table 3:** Correlation study between quality of life Index and clinical characteristics in individuals with CdLS. *Significance level p < 0.05. **Significance level p < 0.01. ID: intellectual disability; GERD: gastroesophageal reflux disease; OPHTH: ophthalmologic. Spearman's correlation coefficients (n = 33).

	Age (years)	Clinical score	Support needs	Intellectual disability	Communication difficulties	Behavior impairment	GER	Limb malformation	Cardiopathy	Seizures	OPHTH defects	Hearing loss
Age (years)	--											
Clinical score	-0.345*	--										
Support needs	0.251	0.294	--									
Intellectual disability	0.194	0.459**	0.427*	--								
Communication difficulties	0.057	0.353*	0.620**	0.648**	--							
Behavior impairment	0.398*	0.512**	0.363*	0.223	0.478**	--						
GERD	0.033	0.407*	0.180	0.281	0.436*	0.442**	--					
Limb malformation	0.109	0.430*	0.297	0.244	0.137	0.151	0.200	--				
Cardiopathy	0.115	-0.214	-0.176	-0.123	0.161	-0.059	0.003	-0.346*	--			
Seizures	0.353*	-0.072	0.358*	0.186	0.212	-0.009	-0.138	-0.058	0.239	--		
OPHTH defects	0.324	0.210	0.200	0.391*	0.219	0.136	0.320	0.242	0.306	-0.306	--	
Hearing loss	-0.143	0.007	-0.264	-0.012	-0.145	-0.013	0.051	-0.068	-0.102	-0.151	-0.210	--
Quality of life index	-0.234	-0.412*	-0.261	-0.136	-0.478**	-0.560**	-0.254	0.025	-0.128	-0.099	0.178	-0.071

When investigating the impact of various risk factors on different domains of QoL and the overall QoLI, we observed that a higher clinical score was associated with lower standardized scores across the domains of social inclusion, self-determination, emotional well-being, physical well-being, rights, and interpersonal relationships, as well as in the total QoLI. Increased support needs were linked to lower scores in the domains of social inclusion, self-determination, and rights. Additionally, heightened communication difficulties and behavioral impairment were associated with lower scores in the domains of social inclusion, self-determination, interpersonal relationships, and the overall QoLI (Table 4).

**Table 4:** Standard scores in different domains and values of the quality of life index according to the different risk factors (n=33). Standard scores in different domains range from 1 to 18. Quality of Life Index ranges from 48 to 130. Values expressed in medium± standard deviation. In bold, significant values ​​between the different degrees of each variable with p<0.05 in the Kruskal-Wallis test. x̄: mean; SD: standard deviation; ID: intellectual disability; GERD: gastroesophageal reflux disease; OPHTH: ophthalmologic; QoLI: quality of life index.

	Social inclusion (x̄ ± SD)	Self-determination (x̄ ± SD)	Emotional well-being (x̄ ± SD)	Physical well-being (x̄ ± SD)	Material well-being (x̄ ± SD)	Rights (x̄ ± SD)	Personal development (x̄ ± SD)	Interpersonal relations (x̄ ± SD)	QoLI (x̄ ± SD)
Gender									
Male	9.9 ± 4.6	10.5 ± 4.8	9 ± 3.7	8.2 ± 4.1	9.9 ± 4.1	10.3 ± 4.4	9 ± 4	10.1 ± 4.3	97 ± 24.1
Female	9 ± 3.4	8.4 ± 3.6	9.4 ± 2.9	9.5 ± 3.2	10.9 ± 2.5	10.2 ± 2.8	9.2 ± 2.7	9.2 ± 2.8	96.4 ± 13
Affected gene									
NIPBL	8.4 ± 4.2	8.8 ± 4.6	8.6 ± 3.1	8.4 ± 3.6	9.8 ± 3.6	9.9 ± 4	8.7 ± 3.3	9.4 ± 3.6	93 ± 19.8
HDAC8	11.7 ± 2.6	12.2 ± 4.5	11 ± 4.7	9.7 ± 4.6	11 ± 3.4	9.3 ± 2.5	10 ± 3.7	11.2 ± 4.2	105.5 ± 21.1
RAD21	11.7 ± 1.5	11 ± 2	7.7 ± 2.1	8.7 ± 4	10.7 ± 1.5	11.7 ± 1.5	7 ± 2.6	9.7 ± 3.1	98.3 ± 9.3
SMC1A	11.3 ± 3.1	7.3 ± 2.5	12 ± 1	10.7 ± 3.2	12.7 ± 0.6	11.3 ± 2.1	12 ± 1.7	8 ± 3.5	104.3 ± 4.7
Unidentified	8.3 ± 3.2	7.7 ± 2.1	10.3 ± 0.6	10.7 ± 2.5	12.3 ± 1.2	11 ± 2.6	10.3 ± 2.1	9 ± 1.7	99.7 ± 2.1
Clinical score									
<9	11.1 ± 2.9	12.4 ± 3.7	11.4 ± 2.4	11.3 ± 2.4	12.4 ± 1.3	12.6 ± 1.7	10.9 ± 3.1	12.4 ± 2.2	111.8 ± 9.6
9-10	11.5 ± 1.7	10.3 ± 4.3	8.5 ± 3.6	6.5 ± 3.7	9.8 ± 3.9	8.8 ± 3.2	9.0 ± 3.5	9.8 ± 3.8	95.2 ± 19.5
>11	7.8 ± 4.1	7.1 ± 3.1	8.4 ± 2.9	8.7 ± 3.3	9.8 ± 3.3	9.6 ± 3.6	8.3 ± 2.9	7.9 ± 2.7	89.5 ± 15.6
Support needs									
Limited	12.6 ± 2.3	13.6 ± 3,2	10 ± 3.4	9.6 ± 3.8	11.6 ± 1.7	13 ± 1.9	10.2 ± 4.4	12.6 ± 3.3	110.2 ± 16.3
Intermittent	11.1 ± 4.3	9.9 ± 3.5	9.9 ± 3.3	8.3 ± 4.2	10.3 ± 3.8	9.7 ± 1.5	9.7 ± 3.2	10 ± 2.6	99 ± 19.3
Extensive	4.2 ± 1.4	7.4 ± 3.1	7.9 ± 3.4	7.8 ± 3.3	9.1 ± 3.9	8 ± 4.4	8.6 ± 3.6	8.4 ± 3.7	88 ± 19.8
Generalized	7.6 ± 2.8	9.4 ± 3.8	9.6 ± 2.8	10.1 ± 3.1	11.2 ± 2.6	11.1 ± 2.8	8.8 ± 2.5	8.7 ± 3	96 ± 12.5
Intellectual disability									
Mild	11.2 ± 4.3	11.7 ± 4.2	8.7 ± 3.2	9.0 ± 4.8	10.0 ± 3.3	11.7 ± 4.1	8.3 ± 4.2	10.8 ± 3.9	100.2 ± 21.5
Moderate	9.5 ± 4.0	9.7 ± 3.9	9.4 ± 3.3	8.3 ± 3.4	10.4 ± 3.9	10.5 ± 2.6	9.2 ± 3.4	10.2 ± 3.5	97.6 ± 20
Severe	8.4 ± 3.3	7.4 ± 3.9	9.3 ± 3.1	9.8 ± 3.0	9.3 ± 3.7	9.3 ± 3.7	9.5 ± 2.4	8.1 ± 2.6	93.9 ± 13.3
Communication difficulties									
Normal	14.5 ± 0.7	16.0 ± 2.8	12.5 ± 2.1	13.5 ± 0.7	13.0 ± 0.1	14.5 ± 0.7	14.0 ± 0.1	15.0 ± 0.1	125.5 ± 3.5
Mild	9.8 ± 5.6	9.3 ± 2.2	9.3 ± 3.1	7.0 ± 4.0	9.8 ± 4.6	11.5 ± 1.7	9.3 ± 3.8	10.8 ± 3.6	97 ± 19.9
Moderate	10.5 ± 2.6	11.1 ± 3.6	11.1 ± 3.6	8.9 ± 3.2	10.9 ± 2.6	10.0 ± 3.6	9.1 ± 2.9	10.5 ± 2.7	100.1 ± 14.6
Severe	7.0 ± 3.4	5.6 ± 2.1	5.6 ± 2.1	9.1 ± 3.6	9.9 ± 3.6	9.5 ± 3.3	8.3 ± 3.2	6.9 ± 2.2	96.6 ± 17.5
Behavior impairment									
Normal	12.3 ± 3.8	13.0 ± 5.6	12.7 ± 1.5	13.0 ± 1.0	13.0 ± 0.1	14.0 ± 1.0	14.0 ± 1.0	13.3 ± 2.9	119.7 ± 10.4
Mild	11.1 ± 2.9	10.8 ± 3.5	9.9 ± 2.4	9.2 ± 2.8	11.3 ± 2.4	9.8 ± 3.6	9.8 ± 2.1	10.3 ± 2.9	101.8 ± 11.4
Moderate	7.9 ± 3.8	7.8 ± 3.6	8.0 ± 3.6	7.8 ± 4.3	9.2 ± 4.1	9.9 ± 2.9	7.9 ± 3.6	8.4 ± 3.7	89 ± 20.3
Severe	5.3 ± 2.1	4.8 ± 1.9	8.0 ± 2.8	9.0 ± 2.6	9.8 ± 2.0	10.0 ± 4.3	7.0 ± 1.6	7.3 ± 0.5	84 ± 4.5
GERD									
Normal	7.5 ± 0.7	7.5 ± 0.7	11 ± 2.8	11 ± 1.4	13	13	12 ± 2.8	11.5 ± 2.1	105 ± 4.2
Mild	10.4 ± 3.5	10.3 ± 3.5	9.6 ± 3	8.7 ± 3.8	10.3 ± 3.4	10.2 ± 3.5	9.4 ± 3	9.9 ± 3.1	99.2 ± 16.6
Moderate	7.9 ± 4.6	8.9 ± 5.6	7.9 ± 3.7	8.4 ± 3.5	9.7 ± 3.3	9.7 ± 3.3	7.7 ± 4.2	9.4 ± 4.5	90.9 ± 24.1
Severe	7.7 ± 4.3	4.5 ± 1.7	8.8 ± 3.2	10.5 ± 3.3	9.7 ± 4.4	9.7 ± 4.3	8.8 ± 1.7	6.7 ± 1.9	89.8 ± 10.9
Limb malformation									
Normal	8 ± 4	9.5 ± 4.5	8 ± 3.7	7.7 ± 4.3	9.4 ± 4.8	11 ± 3.1	7.6 ± 4.1	9.4 ± 4.3	92.2 ± 23.3
Small hand/feet	9.6 ± 3.6	9.4 ± 4.4	9.8 ± 3.1	9.7 ± 3	11 ± 2.2	10.3 ± 3.6	9.9 ± 2.9	10.1 ± 3.1	99.6 ± 16.2
Reduction defect	10.8 ± 4.8	7.4 ± 2.4	9 ± 2.3	8 ± 3.9	10.2 ± 3.6	8.8 ± 2.8	8.4 ± 2.1	7.4 ± 2.2	91.4 ± 11.8
Cardiopathy									
No	9.7 ± 3.6	9.3 ± 4.2	9.4 ± 3.1	9 ± 3.4	10.7 ± 2.7	10.4 ± 3.1	9.2 ± 2.9	9.5 ± 3.1	97.5 ± 15.2
Yes	7.4 ± 4.9	8.2 ± 4.4	8.4 ± 3.6	9.2 ± 4.7	9.2 ± 5.2	9.6 ± 5.2	8.8 ± 4.8	9.4 ± 5	91.4 ± 29.1
Seizures									
No	9.7 ± 3.84.1	9.6 ± 4.1	9.1 ± 3	8.8 ± 3.5	10.6 ± 2.8	10.3 ± 3.4	9.3 ± 2.9	9.8 ± 3	97.4 ± 16.3
Yes	7.7 ± 4.1	7.3 ± 4.1	9.7 ± 4.1	9.8 ± 3.9	10.3 ± 4.7	10.2 ± 3.4	8.5 ± 4.4	8.3 ± 4.8	93 ± 23.6
OPHTH defects									
Normal	10.2 ± 4.1	8.8 ± 3.5	8.2 ± 3	7.9 ± 3.4	10.3 ± 3,2	10 ± 3.5	8.7 ± 3	9.3 ± 2.8	94.4 ± 15.5
Myopia	8.8 ± 3.5	10 ± 4.5	10.9 ± 2.3	9.7 ± 3.2	10.8 ± 2.3	9.9 ± 3	10 ± 2.4	10 ± 3.6	100.4 ± 15.9
Hearing loss									
Normal	9.1 ± 3.8	9 ± 3.8	9.7 ± 2.8	9.1 ± 3.2	10.9 ± 3.2	10.4 ± 3.1	9.5 ± 3.2	9.8 ± 3.2	97.5 ± 17.1
Unilateral	9.7 ± 3.6	9.5 ± 5.4	7.5 ± 3.3	8.7 ± 1	10.7 ± 1.5	9.7 ± 5.9	8.3 ± 2.9	10.2 ± 3.2	95.5 ± 12.9
Bilateral	9.9 ± 4.5	9.4 ± 5.5.1	9 ± 3.9	9 ± 5.1	9.5 ± 3.7	10.1 ± 2.9	8.7 ± 3.5	8.2 ± 3.9	94.8 ± 21.9

**Figure 2:**
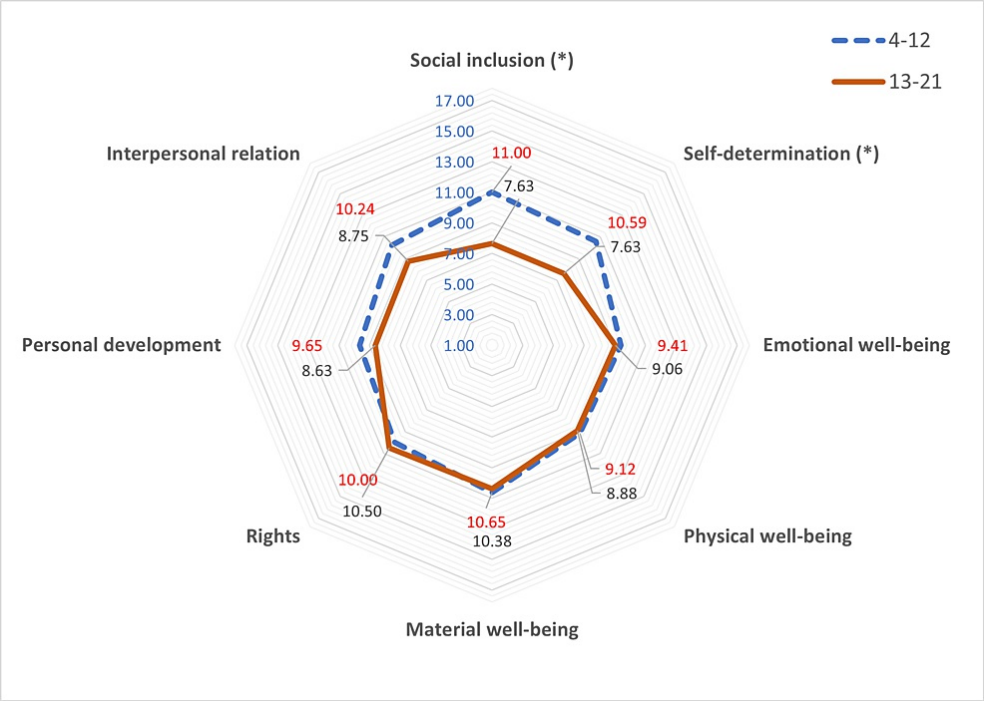
Mean values in standard scores achieved in the eight domains of quality of life according to the age group (4-12 vs. 13-21).

**Figure 3:**
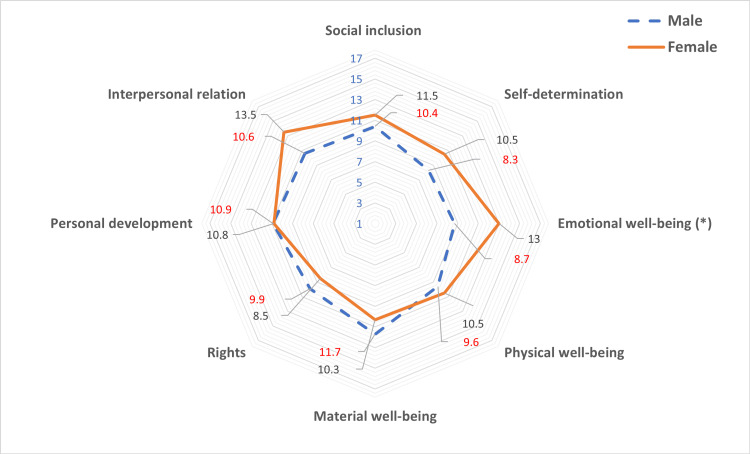
Average values of standard scores achieved in the eight domains of quality of life, stratified by age and sex (male and female).

When categorizing the sample into two age groups (4-12 and 13-21 years), we noted lower scores in the domains of social inclusion and self-determination in the older age group (Figure 2). Finally, upon stratifying the sample by sex, differences were observed in the domain of emotional well-being, with lower values noted in males. However, we found no significant differences in overall quality of life or any of its domains between males and females (Figure 3).

## Discussion

CdLS has been extensively studied from both clinical and molecular perspectives; however, this study represents the first focused on assessing the QoL of affected individuals. Given the absence of available etiological treatments, it is essential to channel our efforts towards minimizing the consequences of the disease, taking into consideration not only physical aspects but also social and emotional factors [25].

Several questionnaires exist for evaluating the QoL in children and adolescents with intellectual disabilities [19-21]. For our study, we opted for the Kidslife scale [22] due to its comprehensive coverage of various domains, suitability for the age range of our cohort (4 to 21 years), and its non-specific nature to any condition. This user-friendly questionnaire can be completed by parents, caregivers, or legal guardians. Moreover, it has demonstrated successful application in individuals with other genetic syndromes, such as Down syndrome [26] and Williams syndrome [27].

The average QoL within our cohort is situated at the 45.3rd percentile, a value below the median observed in individuals with other intellectual disabilities and notably lower than the results reported in Down syndrome (70-71st percentile) [26]. This disparity can be attributed to the lower social visibility of CdLS and the more pronounced physical, cognitive, and behavioral impairments compared to other genetic syndromes [28-30]. Despite advancements in social awareness, inclusive practices, medical treatments, and educational and employment support for individuals with CdLS, these findings underscore the significance of implementing additional measures.

In the analysis of the eight domains of QoL, elevated scores are particularly observed in the domains of material well-being and rights. The material well-being domain encompasses the capacity to meet fundamental needs, incorporating assistive technologies to enhance autonomy, essential material goods for daily life, and the availability of an environment, housing, and educational facilities that are adapted. A higher score within this domain may suggest that society allocates resources to ensure these services. However, it is crucial to underline that families of individuals with CdLS allocate a substantial portion of their income to fulfill these needs and secure a fulfilling life for their children. As a society, it is imperative to explore avenues for providing enhanced support to these families. The rights domain pertains to the respect for individual rights, possessions, privacy, and confidentiality of their assessments. It also considers whether individuals are informed about decisions made on their behalf and if their participation in activities is facilitated with the same opportunities as others. A higher score in this domain may indicate the existence of measures ensuring their rights and fostering their inclusion in society. However, despite achieving the highest scores, it is noteworthy that the values obtained remain comparatively low in comparison to other intellectual disabilities.

The lowest scores are in the domains of physical well-being, self-determination, and personal development. The domain of physical well-being refers to nutrition, care of appearance, physical activity, medical care, and preventive health measures. In CdLS, unlike other genetic syndromes associated with intellectual disability and behavioral alterations, affected individuals may present significant physical malformations that directly impact their physical well-being, personal development, and autonomy. A low score in this domain may result from the extensive multisystemic involvement of individuals with CdLS, as well as the lack of knowledge about the syndrome among healthcare professionals. This underscores the significance of establishing reference centers staffed by professionals well-versed in the syndrome.

On the other hand, the domain of self-determination refers to providing the necessary means for individuals to make their own decisions and take their opinions into account when considering possible changes. The lack of self-determination negatively impacts QoL, so it is necessary to continue working on the development of tools that enable individuals with CdLS to actively participate in choices that affect them. In this regard, the use of tablets or pictograms has been of great assistance. The domain of personal development focuses on the presence of measures that promote learning ability, skills, and independence. A low score in this domain may indicate the need for better-trained caregivers, more specific educational programs, and educational materials that are better adapted to their abilities.

Families involved with the CdLS association benefit from resources and support they might not have access to individually. Furthermore, the Spanish healthcare system provides universal coverage for necessary medical care. To identify risk factors, the sample has been divided into three groups based on low, medium, and high QoL. The results suggest that individuals with a mutation in the NIPBL gene and/or a clinical score greater than 11 (indicative of a classical phenotype) and/or severe behavioral problems have poorer QoL. This clinical score is a diagnostic tool that classifies affected individuals by evaluating physical and cognitive characteristics [1]. The classical phenotype is more common in individuals with mutations in the NIPBL gene, which is also often associated with a more problematic behavioral profile [31].

In the study conducted on the total sample, we observed a negative correlation between the clinical score, communication problems, behavioral problems, and QoL. The clinical score was identified as a factor that negatively affects most domains, except for material well-being and personal development. Individuals with CdLS who experience greater communication and behavioral problems have reduced scores in the domains of social inclusion, self-determination, and interpersonal relationships, possibly due to difficulties in being accepted by society and expressing their own choices. Furthermore, communication problems play a significant role in the emergence of behavioral disorders, such as self-injury or challenging behaviors, which are related to the inability to express somatic complaints and issues [32, 33]. Individuals with higher support needs also experience significant effects on the domains of social inclusion, self-determination, and rights.

It is interesting to note that the degree of intellectual disability does not appear to influence overall QoL or its respective domains, mirroring observations in Williams syndrome [27]. Neither sex, GERD, skeletal malformations, presence of heart disease, epilepsy, nor visual or auditory problems seem to have a significant impact. Despite the previously documented association between GERD and deteriorated behavior, it is important to emphasize that individuals within our cohort received adequate treatment [34, 35].

A notable decline in social inclusion and self-determination is observed during adolescence. This may be attributed to the increased occurrence of behavioral and communication disorders during this stage, although it tends to improve after the age of 20 years [31]. The reviewed studies addressing behavior in CdLS employ heterogeneous methods of assessment, indicating the need for a tool that allows for more standardized evaluation [36].

One limitation of this study is its limited sample size. However, it is important to note that CdLS is a rare disease with a very low prevalence in the general population. Our sample, despite being small, is representative of CdLS-diagnosed patients in Spain. Families with members diagnosed with CdLS are referred to the association. Another consideration is that the questionnaire responses are based on the perceived QoL obtained from parents.

## Conclusions

In conclusion, this study represents the first comprehensive assessment of the QoL in individuals affected by CdLS. The selection of the Kidslife scale for QoL evaluation was based on its broad coverage across various domains, suitability for the age range of our cohort, and applicability to individuals with diverse genetic syndromes. Individuals with CdLS exhibit lower QoL compared to those with other intellectual disabilities, attributed to the syndrome's lower social visibility and pronounced physical, cognitive, and behavioral impairments.

Factors such as a classical phenotype associated with NIPBL gene mutations, as well as severe communication and behavioral problems, are identified as significant determinants impacting QoL. It is crucial to identify and address modifiable risk factors to establish specific interventions. Considering the clinical score, some of these interventions would include the correct identification and treatment of hiatal hernia, orthopedic measures to minimize the consequences of limb malformation, and early stimulation in specialized centers to address developmental delays. Regarding behavioral problems, it is important to determine the presence of underlying causes such as pain or GERD and address them properly. Once organic causes have been ruled out, appropriate psychological and medical therapies should be considered to address the specific behavioral disorders identified. Lastly, it is essential to provide applicable communication tools to enable individuals to express their needs and desires and to promote their participation in social interactions.

Finally, we highlight the need for a comprehensive approach to the management of CdLS, addressing clinical, molecular, psychosocial, and emotional aspects. The evaluation of QoL and the identification of risk factors stand out as invaluable tools for comprehending current needs and implementing interventions focused on enhancing overall well-being.
